# Statistical Approach for Optimization of Physiochemical Requirements on Alkaline Protease Production from *Bacillus licheniformis NCIM 2042*

**DOI:** 10.1155/2012/905804

**Published:** 2012-01-05

**Authors:** Biswanath Bhunia, Apurba Dey

**Affiliations:** Department of Biotechnology, National Institute of Technology, Mahatma Gandhi Avenue, Durgapur 713209, India

## Abstract

The optimization of physiochemical parameters for alkaline protease production using *Bacillus licheniformis NCIM 2042* were carried out by Plackett-Burman design and response surface methodology (RSM). The model was validated experimentally and the maximum protease production was found 315.28 U using optimum culture conditions. The protease was purified using ammonium sulphate (60%) precipitation technique. The HPLC analysis of dialyzed sample showed that the retention time is 1.84 min with 73.5% purity. This enzyme retained more than 92% of its initial activity after preincubation for 30 min at 37°C in the presence of 25% v/v DMSO, methanol, ethanol, ACN, 2-propanol, benzene, toluene, and hexane. In addition, partially purified enzyme showed remarkable stability for 60 min at room temperature, in the presence of anionic detergent (Tween-80 and Triton X-100), surfactant (SDS), bleaching agent (sodium perborate and hydrogen peroxide), and anti-redeposition agents (Na_2_CMC, Na_2_CO_3_). Purified enzyme containing 10% w/v PEG 4000 showed better thermal, surfactant, and local detergent stability.

## 1. Introduction

The protease is ubiquitous in nature. It is found in all living organisms and required for cell growth and differentiation. Alkaline proteases are one of the most important groups of industrial enzymes. They are extensively used in leather, food, pharmaceutical, textile, organic chemical synthesis, wastewater treatment, and other industries [1]. Alkaline proteases hold a major share of the enzyme market with two-third share in detergent industry alone [2, 3]. Due to enhancement of such demand of proteases for specific properties, scientists are looking for newer sources of proteases. For effective use in industries, alkaline proteases need to be stable and active at high temperature and pH and in the presence of surfactants, oxidizing agents, and organic solvents [4–7]. Although there are many microbial sources available for protease production, only a few are considered as commercial producers [8]. Of these, species of *Bacillus *dominate in the industry [9].

Only large-scale production of alkaline protease can fulfill the demand and usefulness of the proteases in the industry. In industry, microbial protease production was carried out by fermentative process. It is necessary to improve the yield of protease without increasing the process cost through fermentative process. Rapid enzyme production can be achieved by manipulation of media composition and culture conditions. Thus, optimization of fermentation conditions is the most important step in the development of a cost-effective fermentation process [10]. In our previous study, we reported partial characterization of serine protease from *Bacillus licheniformis NCIM 2042*. Maximum enzyme activity was found to be at pH 9.0, temperature 75°C. The enzyme was stable at 50°C for 1 h, over a broad pH range (6.0–12.0) and in the presence of H_2_O_2_, SDS, Triton X-100, DMSO, methanol, ethanol, ACN, and 2-propanol [7].

In this study, a systematic and sequential optimization strategy was applied to enhance the production of alkaline protease from *Bacillus licheniformis NCIM 2042*. Screening of the significant variables was carried out by 2-level factorial designs using the Plackett-Burman design. Then, optimization of the screened variables was carried out by response surface methodology (RSM) for extracellular alkaline protease production from *Bacillus sp*. Furthermore, various attempts have been made to enhance stability of partial purified enzyme using different additives.

## 2. Materials and Methods

### 2.1. Chemicals and Analysis

Bradford Reagent (Sigma, USA), bovine serum albumin (BSA) (Himedia, India), Dialysis sacks (Sigma, USA), Methanol for HPLC (E-Merck, Germany), Acetonitrile for HPLC (E-Merck, Germany), Triton X-100 (E-Merck, Germany), 2-propanol (Merck, India), benzene (Merck, India), toluene (Merck, India), hexane (Merck, India), Tween-80 (Merck, India), PEG 4000 (Merck, India), PEG 600 (Merck, India), Trichloroacetic acid (Merck, India), Mannitol (Himedia, India), Glycerol (Himedia, India), SDS (Himedia, India), DMSO (Himedia, India), and Casein (Himedia, India) were used in this study. Water used for the HPLC analysis was prepared by Ultrapure Water System (Arium, 611UF, Sartorius, Germany). All other chemicals used were of analytical grade and commercially available in India. The statistical software package “Design Expert” 7.0.0 (Stat-Ease Inc., Minneapolis, USA) was used to analyze the experimental design and the regression analysis of the experimental data.

### 2.2. Microorganism and Seed Culture

The protease producing *Bacillus licheniformis NCIM 2042* was procured from NCL, Pune, India, and grown on nutrient agar slants at 37°C at pH 7.4. It was maintained by subculturing on nutrient agar slants kept at pH 7.4. For production experiments, the culture was revived by adding a loop full of pure culture into 50 mL of sterile nutrient broth (pH 7.4).

### 2.3. Protease Production

A 2% fresh culture (OD_550_ *≈* 0.2) was inoculated in 50 mL complex media of 250 mL Erlenmeyer flask, containing optimized media (gl^−1^); starch, 30.8; soybean meal, 78.89; K_2_HPO_4_, 3; KH_2_PO_4_, 1; MgSO_4_, 0.5; NaCl, 5.27. The culture was centrifuged at 10,000×g for 10 min at 4°C. The cell pellet was discarded and the supernatant was used for assay of protease activity. 

### 2.4. Enzyme Assay and Determination of Protein Concentration

Protease activity was determined by a modified method of Folin and Ciocalteu [7]. Protein concentration was determined by the method of Bradford using bovine serum albumin (BSA) as the standard [11]. All the experiments were done in triplicate.

### 2.5. Optimization of Alkaline Protease Production

#### 2.5.1. Selection of Physical Parameter

Inoculum percentage, temperature, pH, agitation, and incubation time [12–14] are considered to contribute alkaline protease production from *Bacillus sp*.

#### 2.5.2. Screening of Significant Media Components by Plackett-Burman Factorial Design

The screening of the physical parameters was done by Plackett-Burman design with respect to their main effects and not to their interaction effects [15]. For the alkaline protease production, five factors were selected, namely, inoculum percentage (*A*), temperature (*B*), pH (*C*), agitation (*D*), and incubation time (*E*). The effect of five factors on protease production was studied using statistical approach. Each parameter was experimented at two levels, (high and low), which was decided from previous unreported work. A set of 12 experiments was carried out to determine alkaline protease production under different combinations as given in Table 1. The effect was calculated by changing the response as the factor changes from its lower (−1) level to its higher (+1) level using student's *t*-test. The *P* value of individual variables was also evaluated. The variables with *P* values less then 0.05 (*P* value <0.05) were considered as significant factors for protease production.

#### 2.5.3. Optimization of Key Determinants by Response Surface Methodology

RSM is employed for multiple regression analysis. It solves polynomial equations using quantitative data obtained from properly designed experiments [16, 17]. In this experiment RSM was used to determine the optimum culture condition for the production of alkaline protease. A central composite rotatable design (CCRD) was used for five independent variables to obtain optimum value. Inoculum percentage, temperature, pH, agitation, and incubation time were selected as the physical parameters (independent variables) for the optimization. Regression analysis was performed on the data obtained from the design experiments. A set of 50 experiments including eight center points was carried out. Each numeric factor is varied over 5 levels (−2.38, −1, 0, +1, +2.38), that is, plus and minus *alpha* (axial point), plus and minus one (factorial points), and zero (center point). The full experimental plan with respect to their actual and coded forms is listed in Table 2. The response values (*Y*) in each trial were the average of the triplicates.

Analysis of variance (ANOVA) was used for analysis of regression coefficient, prediction equations, and case statistics. The experimental results of RSM were fitted via the response surface regression procedure, using the following second order polynomial equation:

(1) $$Y=\begin{array}{cccc} \beta_{o} & +\sum \beta_{i}X & +\sum \beta_{ii}Xi^{2} & +\sum \beta_{ij}XiXj.\\ & i & ii & ij \end{array}$$

In this polynomial equation, *Y* is the predicted response, *X*_*i*_ *X*_*j*_ are independent variables, *β*_*o*_ is the intercept term, *β*_*i*_ is the linear coefficient, *β*_*ii*_ is the quadratic coefficient, and *β*_*ij*_ is the interaction coefficient.

However, in this study, the independent variables were coded as *A*, *B*, *C*, *D*, and *E*. Thus, the second-order polynomial equation can be represented as follows:

(2) $$\begin{array}{cc} Y & =\beta_{o}+\beta_{1}A+\beta_{2}B+\beta_{3}C+\beta_{4}D+\beta_{5}E\\ & \;+\beta_{11}A^{2}+\beta_{22}B^{2}+\beta_{33}C^{2}+\beta_{44}D^{2}\\ & \;+\beta_{55}E^{2}+\beta_{12}AB+\beta_{13}AC+\beta_{14}AD\\ & \;+\beta_{15}AE+\beta_{23}BC+\beta_{24}BD+\beta_{25}BE\\ & \;+\beta_{34}CD+\beta_{35}CE+\beta_{45}DE. \end{array}$$

The second-order polynomial coefficients and Response surface plots were obtained using the Design Expert software.

#### 2.5.4. Validation of the Experimental Model

The statistical model was validated with respect to all the three variables within the design space. A random set of 6 experimental combinations was used to study protease production in 250 mL shake flasks.

### 2.6. Partial Purification and Characterization

The fermented broth was centrifuged at 10000 rpm for 30 min at 4°C. At first, solid ammonium sulphate was added to the supernatant for 30% saturation and centrifuged at 4°C for 30 min. Again, solid ammonium sulphate was added to the supernatant for 60% saturation and centrifuged at 4°C for 30 min. The precipitate was resuspended in 50 mM phosphate buffer (pH 9). The precipitated sample was desalted by dialysis through semipermeable membrane (molecular weight cutoff 12 kD, Sigma) against same buffer for overnight.

#### 2.6.1. Organic Solvent Stability of Partially Purified Protease

Stability of the enzyme in organic solvents was studied by incubating the enzyme solution (3 mL) with various organic solvents (1 mL), namely, DMSO, methanol, ethanol, ACN, 2-propanol, benzene, toluene, and hexane at 37°C with shaking at 150 rpm for 30 min. The remaining activity of the enzyme was measured. The activity of the enzyme solution without organic solvent was considered as control (100%).

#### 2.6.2. Effect of Nonionic Detergent, Surfactants, and Bleach Agents on Partially Purified Protease

The enzyme was incubated with some nonionic detergents (5% v/v Tween-20, Tween-80, and Triton X-100), surfactants (0.5% w/v SDS), bleaches (5% v/v H_2_O_2_, 0.5% w/v sodium perborate), and anti-redeposition agents (10% w/v Na_2_CMC, 100 mM Na_2_CO_3_) for 60 min at room temperature and remaining activity was measured. The activity of the enzyme solution without modifier was considered as control (100%).

#### 2.6.3. Effect of Different Additives on Thermal Stability of Partially Purified Enzyme

Thermal stability was determined by incubating the partially purified enzyme at 60°C (pH 9), in the presence of 2.5 and 5 mM CaCl_2_ and in the presence of various polyols (5, 10% w/v). Polyols used in this study were PEG-4000, PEG-600, mannitol, and glycerol. Aliquots were withdrawn at 15 min intervals and the residual activity was determined. The activity of the enzyme solution kept at 4°C was considered as control (100%).

#### 2.6.4. Effect of Polyols on Surfactant Stability

The surfactant stability was determined by incubating the partial purified enzyme in the presence of surfactants (0.5% w/v SDS) and in the presence or absence of various polyols (10% w/v) for 60 min at room temperature. Polyols used in this study were PEG-4000, PEG-600, mannitol, and glycerol. Remaining activity of the enzyme was measured. The activity of the enzyme solution without modifier was considered as control (100%).

#### 2.6.5. Effect of Polyols on Detergent Stability

For detergent stability study, the purified alkaline protease (0.05 mg/mL) was incubated at 40°C using Surf excel (7 mg/mL) in the presence of various polyols (10% w/v). At every 15 min interval, the residual protease activity was determined up to 1 hour. The purified alkaline protease without detergent was considered as control (100%).

#### 2.6.6. Evaluation of Washing Performance Using Blood Stain

Clean cotton cloth pieces were stained with 25 *μ*L of human blood and allowed to dry. The stained-cloth pieces were treated with tap water, Rin (0.7% (w/v), in tap water) and detergent added with partially purified enzyme (2000 U). Each flask was incubated at 50°C for 60 min under agitation (150 rpm). After incubation, the cloth pieces were taken out, rinsed with water, and dried.

### 2.7. HPLC Analysis of Partially Purified Enzyme

Partially purified enzyme was qualitatively analyzed using HPLC. For sample preparation, the sample was centrifuged at 10000 rpm for 10 min at 4°C and the supernatant filtered through 0.45 *μ*m nylon membrane filters. After appropriate dilutions with phosphate buffer, sample was analyzed. For the analysis of purity of sample, Waters-600-Pump-based HPLC system equipped with Waters 2489 UV/Visible Detector was used. Water Empowered software (Version: Empower 2 software Build 2154) was used for data acquisition and mathematical calculations. Chromatographic separation of protease was performed on a C_18_ hypersil column (4.6 mm × 250 mm; 5 *μ*m particle size; Waters, USA). Mobile phase used was acetonitrile water (70 : 30 vv^−1^), at a flow rate of 1 mL/min. Temperature of the column oven was maintained at 30°C. The sample (20 *μ*L) was injected and analyzed at 280 nm using UV-visible detector.

## 3. Result and Discussion

### 3.1. Optimization of Alkaline Protease Production from *Bacillus licheniformis NCIM 2042*

#### 3.1.1. Screening of Significant Physical Parameters

Plackett-Burman design was adopted to select most significant physical components. The studentized effect corresponding sum of square, standard error, percentage of contribution, *t*-value and *P* value are given in Table 3. The experimental design along with the responses of different experimental trials is shown in Table 4. studentized effect allows the determination of the effect of each component. A large Studentized effect either positive or negative indicates that a factor has a large impact on production, while an effect close to zero means that a factor has little or no effect. The *P* value is the probability which serves as a tool for checking the significance of each of the parameter. A low *P*-value indicates a “real” or significant effect. The significance of each variable was determined by applying the Student's *t*-test [18]. In our study, the five key determinants namely inoculum percentage (*A*), temperature (*B*), pH (*C*), agitation (*D*), and incubation time (*E*) having lower *P* value (*P* value < 0.05) were identified to be the significant variables for extracellular protease production.

As Plackett-Burman design is inappropriate to study the mutual interaction of process variables, therefore the level of significant factors needed further optimization. In this investigation, RSM was applied for the optimization of significant factors in protease production to study the importance of screening factors at different levels. The RCCD design plan of RSM was used in the present study and the physicochemical components were optimized for maximum protease production.

#### 3.1.2. Optimization of the Key Determinants

The full experimental plan of CCD design for studying the effects of five independent variables, namely, inoculum percentage (*A*), temperature (*B*), pH (*C*), agitation (*D*), and incubation time (*E*) are listed in Table 5. The statistical significance of the second-order polynomial equation was checked by an *F*-test (ANOVA) and data shown in Table 6. The Model *F*-value of 5602.23 implies the model is significant. It may be mentioned that the probability of obtaining high model *F*-value due to creation of noise is only 0.01%. Values of “prob > *F*” less than 0.0500 indicate model terms are significant. In this case *A*, *B*, *C*, *D*, *E*, *AB*, *AD*, *AE*, *BC*, *BD*, *BE*, *CD*, *CE*, *DE*, *A* ^2^, *B* ^2^, *C* ^2^, *D* ^2^, *E* ^2^ are significant model terms. Moreover, “lack of Fit *F*-value” of 0.07 implies that it is not significant relative to the pure error. Nonsignificant lack of fit indicates a good fitness of model. There is only 100% chance that this magnitude of “Lack of Fit *F*-value” could occur due to noise. The correlation coefficient (*R* ^2^) of polynomial equation was found to be 0.9997 indicating that 99.97% of the variability in the response (alkaline protease production) could be explained by the model. Thus, quadratic model was chosen for this analytical work. The adjusted *R* ^2^ value corrects the *R* ^2^ value for the sample size and for the number of terms in the model. The adjusted *R* ^2^ (0.9996) is also very high that indicates that the model is very significant. The “Pred R-Squared” value of 0.9996 is in reasonable agreement with the “Adj R-Squared” value of 0.9996. This indicated a good adjustment between the observed and predicted values. Adeq Precision” measures the signal to noise ratio. A ratio greater than 4 is desirable. Our ratio of 256.23 indicates an adequate signal. This model can be used to navigate the design space. The coefficient of variation % (CV%) is a measure of residual variation of the data relative to the size of the mean. Usually, the higher the value of CV, the lower is the reliability of experiment. Here a lower value of CV (0.64%) indicates a greater reliability of the experimental performance. The predicted residual sum of squares (PRESS) is a measure of how well the model fits each point in the design. The smaller the PRESS statistic, the better the model fits the data points. Our value of PRESS is 81.03. The model shows standard deviation and mean value of 1.27 and 198.51, respectively.

**Figure 1:**
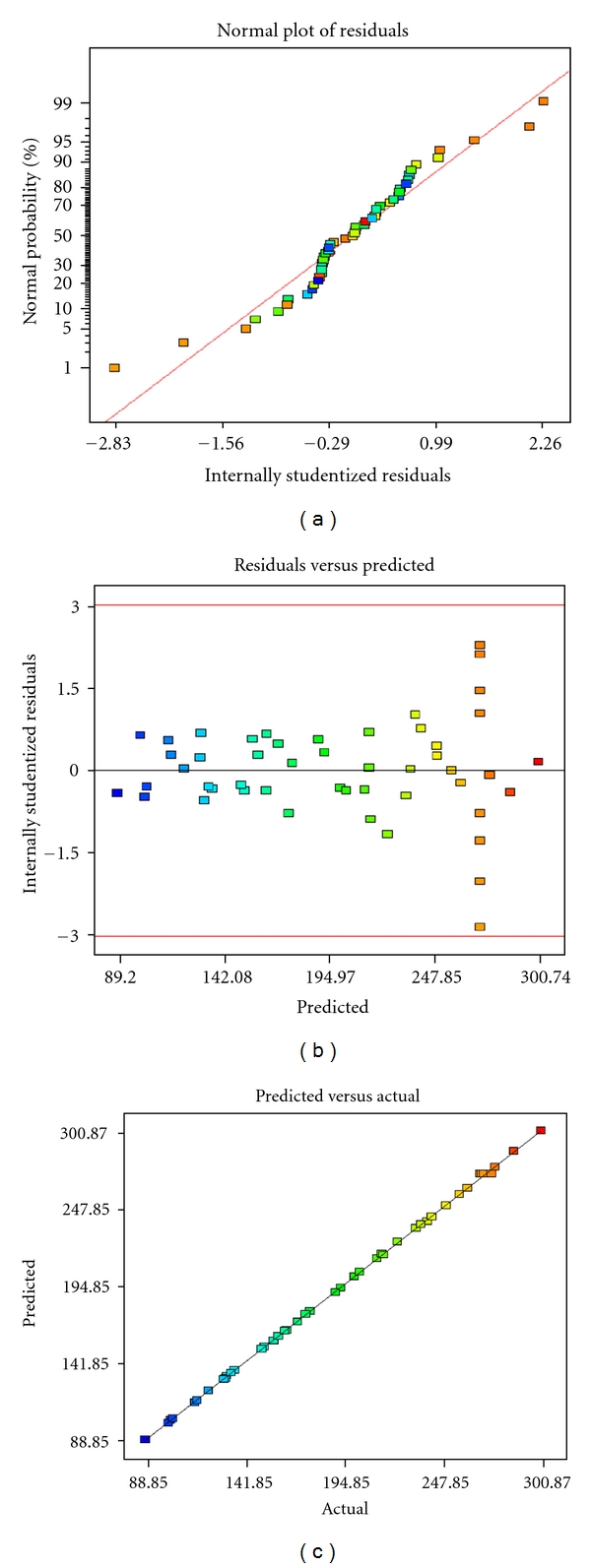
(a) Normal plot of residual; (b) residual versus predicted plot; (c) Predicted versus Actual Response plot for protease production (U) by *Bacillus licheniformis NCIM 2042*.

**Figure 2:**
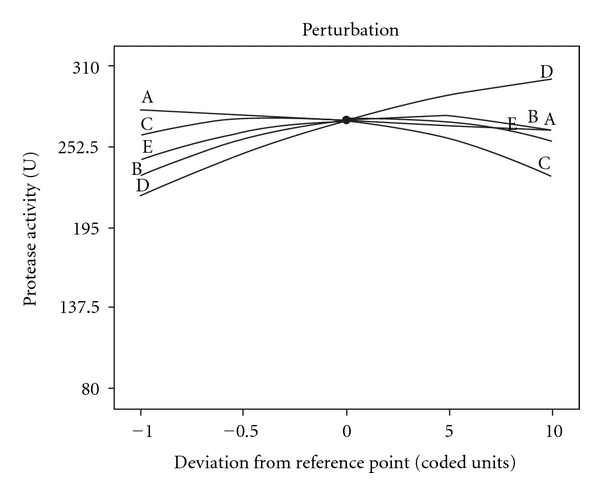
Perturbation plot for the production of alkaline protease by *Bacillus licheniformis NCIM 2042* as a function of inoculum percentage (*A*), temperature (*B*), pH (*C*), agitation (*D*), and incubation time (*E*).

The normal probability plot given in Figure 1(a) shows some scatter along the line indicating that the residuals follow a normal distribution. Residuals versus predicted plot in Figure 1(b) indicate the residuals versus the ascending predicted response values. The plot shows a random scatter (constant range of residuals across the graph). Actual versus predicted plots (Figure 1(c)) also represents a high degree of similarity that was observed between the predicted and experimental values. From the three diagnostic plots (Figures 1(a)–1(c)) it can be concluded that the model has satisfied the assumptions of the analysis of variance. This model also reflected the accuracy and applicability of RSM to optimize the process for protease production. Perturbation plot in Figure 2 represents comparison of the effect of physical parameters at the midpoint (coded 0) in the design space. A curvature curve were found with all factors shows that the response is sensitive to all factors.

**Figure 3:**
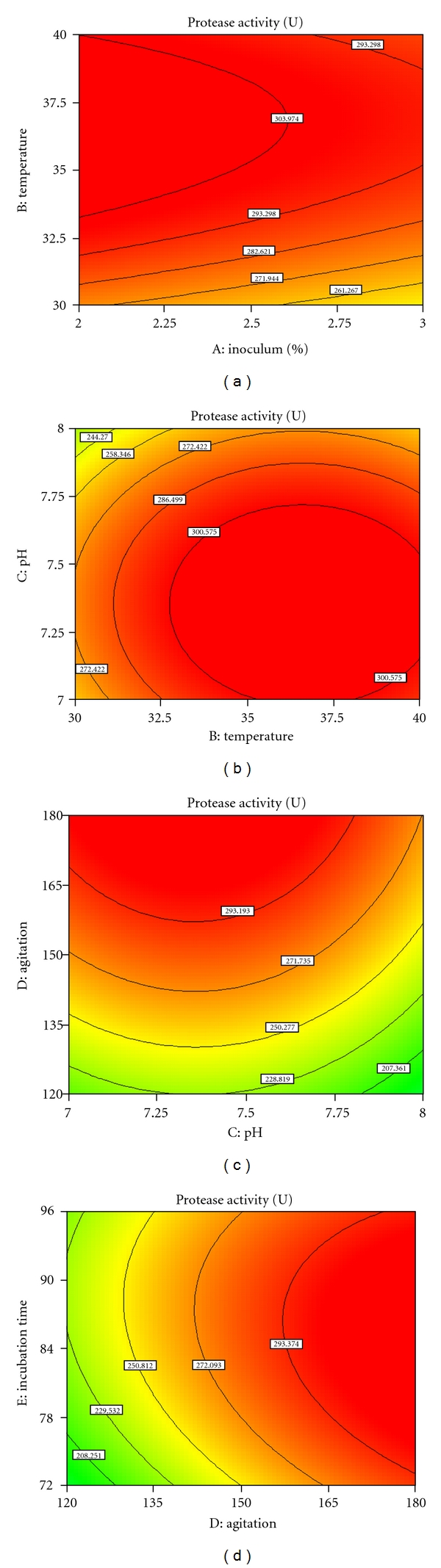
Contour plot for protease production (U) by *Bacillus licheniformis NCIM 2042* as a function of (a) inoculum percentage (*A*) and temperature (*B*), (b) temperature (*B*) and pH, (*C*), (c) pH (*C*) and agitation (*D*), and (d) agitation (*D*), and incubation time (*E*) when other variables are at zero level.

 The special features of the RSM tool are contour plot generation and point prediction from, where we can determine the optimum value of the combination of the five physical parameters for the maximum production of alkaline protease. The contour plot (Figures 3(a)–3(d)) determines the interaction of the physical parameters. These plots were obtained from the pairwise combination of independent factors, while the keeping other factor at its center point level. From the eliptical contour plot, it is clearly indicated that the mutual interaction is prominent among factors. The predicted results from the point prediction method showed that maximum protease production was obtained with optimum inoculum percentage (2%), temperature (36.6°C), pH (7.35), and agitation (180 RPM) after 85 h fermentation in the shake flask. The maximum predictable response was calculated using regression equation by substituting level of factors and was experimentally verified.

 In our previous experiment 184.27 U of protease was found after media optimization (data not shown). A significant improvement (1.71-fold) in the alkaline protease production by *Bacillus licheniformis NCIM 2042 *was found after optimization of culture condition. In this experiment we attempt to optimize the culture condition because each microorganism has its own individual physicochemical and nutritional requirements for growth and enzyme secretion. Accurate process optimization influences the activities of microorganisms and improves the production significantly, which is desirable for minimization of processing cost. In this experiment the maximum production of alkaline protease obtained experimentally using optimization of culture condition was 315.28 U which is likely similar with predicted value (314.65 U) by the model. There are previous reports of optimum pH and temperature conditions for alkaline protease activity from *B. licheniformis NCIM 2042* to be 8 and 30°C, respectively [19, 20]. But a higher optimum pH and temperature of alkaline protease was found in our previous work, which might be of significant industrial importance [7].

#### 3.1.3. Validation of the Experimental Model

The model was validated for all five variables within the design space. A random set of six production combinations was prepared and tested for protease production (given in Table 7). The experimentally determined production values were in close agreement with the statistically predicted ones, confirming the model's authenticity and applicability of the statistical model (RSM) for the optimization of the medium nutrients.

### 3.2. Partial Purification and Characterization of Partially Purified Enzyme

Partial purification of crude enzyme was done by salting out technique using ammonium sulphate as the salt. The specific activity of the partially purified enzyme was found to be 158.52 (U/mg). The purification fold and yield of protease from the crude enzyme solution were 17.34 fold and 82.43%, respectively (data not shown).

#### 3.2.1. Organic Solvent Stability of Partially Purified Protease

The organic solvents are also used as the media for enzymatic reactions. But the enzymes are easily deactivated in organic solvent. Usually, presence of organic solvent reduces the structural flexibility of enzyme which is required for catalysis. There are published reports of protease inactivation in the presence of water immiscible organic solvent (benzene, toluene, xylene, and hexane) [6, 21] and water miscible organic solvents like ethanol, acetone, and DMSO [22, 23]. Therefore, proteases that are stable in the presence of organic solvents are very useful for synthetic reactions. The structural stabilities of protease are highly dependent on the solvent-protein interactions and the enzyme structure [24]. The stability of the enzyme also depends on the chemical composition of organic solvents because solvent polarity is one of the factors determining the stability of biocatalyst [21].

**Figure 4:**
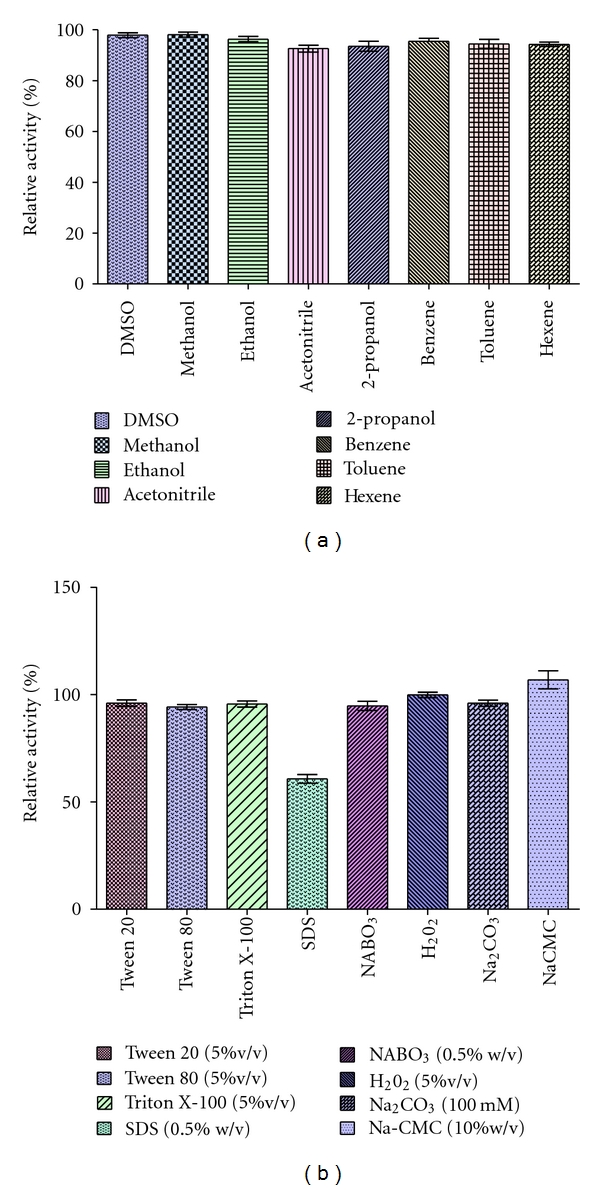
Effect of (a) organic solvent and (b) detergent, surfactant, bleach, and anti-redeposition on protease activity.

The effects of organic solvents on protease activity differ among proteases. Organic solvents with a different log *P* between −1.378 and 3.5 were used in this study. Log *P* is the logarithm of the partition coefficient of a solvent in a defined octanol-water mixture. It is commonly used for measurement of the lipophilicity of a solvent. The effect of different organic solvents on protease activity is shown in Figure 4(a). In the present study, the partially purified protease remained more than 92% of its initial activity after 30 min of preincubation with most of the tested organic solvents. The organic solvent stability was observed in the following order: methanol (log *P* −0.764) > DMSO (log *P* −1.378) > ethanol (log *P* −.235) > benzene (log *P* 2.0) > toluene (log *P* 2.5) > hexane (log *P* 3.5) > 2-propanol (log *P* 0.074) > acetonitrile (log *P* −0.394). The results obtained in the above set of experiments indicate that protease enzyme is mostly stable in water miscible and water immiscible organic solvent up to 30 minutes. So this enzyme can be used for peptide synthesis.

#### 3.2.2. Effect of Oxidizing Agents, Surfactants, and Bleach Agents on Protease

This study showed that the enzyme was more than 94% stable at 5% v/v of non ionic detergent (Tween-80, Triton X-100) and 60% residual activity was obtained after incubation with 0.5% w/v SDS. Such effect variation of detergent can be explained due to their different hydrophilic/lipophilic balance (HLB) number. HLB of detergent is defined as the way a detergent distributes between polar and nonpolar phases [1]. The HLB number of Triton X-100 and Tween-80 is 13.5 and 15. So they are less detrimental as compared to SDS with high HLB number (40). Enzyme was also very stable against bleaching agents since it retained 94.67% and 99.80% of its initial activity after treatment with 0.5% (w/v) sodium perborate and 5% (v/v) hydrogen peroxide, respectively. Such characteristics of protease is an important behaviour because detergent, surfactant, and bleach-stable wild-type enzymes are rarely reported [25]. These results are closely similar with previous reports [1, 26–28]. Besides, the protease was almost stable in the presence of anti-redeposition agents (10% w/v Na_2_CMC, 100 mM Na_2_CO_3_). It is interesting to note that activation of enzyme activity was found in presence of 10% w/v Na_2_CMC which is similar with previous report [25]. From the result, it is clearly indicated that our purified enzyme is mostly stable in the detergent components (Figure 4(b)). Therefore, enzyme could be considered as a potential candidate for use as cleaning additive in detergents.

#### 3.2.3. Effect of Different Additives on Thermal Stability

**Figure 5:**
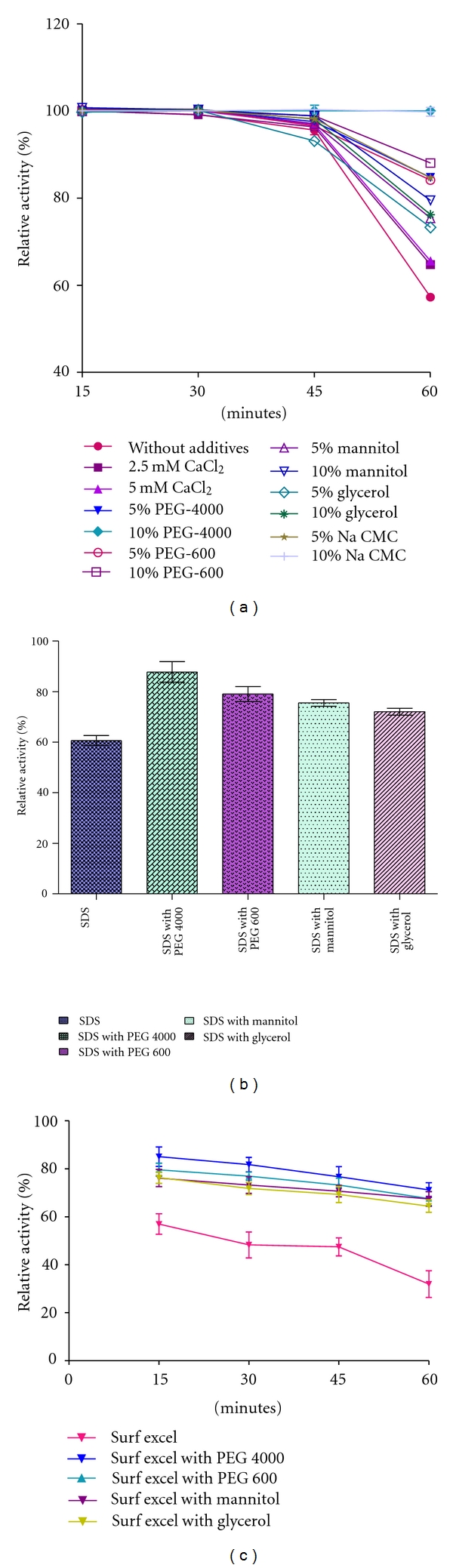
Stabilizing effect of additives on protease stability in presence of (a) high temperature, (b) surfactant, and (c) local detergent.

Thermostability profile of purified enzyme was determined by incubating the enzyme at various temperatures that is, 40, 50, 60, 65, 70, 75, and 80°C for 1 hr. The residual activity of the enzyme was determined at standard assay condition. Results showed that enzyme was stable and retained almost its full activity after 60 min incubation at 40 and 50°C. The enzyme was found to be 57.22, 23.7, 13.45, and 1.07% stable at 60, 70, 75 and 80°C respectively after 60 min incubation (data not shown). So further study was done to evaluate the effect of different additives on thermal stability. The data presented in Figure 5(a) showed that most used additives improve thermal stability. However, thermostabilization was more effective with 10% w/v PEG 4000. Result showed that the enzyme was stable 100, 88, 79.49,76, and 65.59% at 10% w/v of PEG 4000, PEG 600, mannitol, glycerol, and 5 mM calcium chloride, respectively, after 60 min incubation. The protective effect of polyols was explained by the strengthening of the hydrophobic interactions inside protein molecules and by indirect action of polyols on water structure. The improvement of thermostability at higher temperature with respect to incubation time in the presence of Ca^2+^ is due to strengthening of interaction inside protein molecule and binding of Ca^2+^ to autolysis site. These results were similar to earlier reports indicating that the addition of calcium chloride and polyhydric alcohols such as PEG, mannitol, and glycerol increased in thermal stability of alkaline proteases [29, 30].

#### 3.2.4. Effect of Polyols on Surfactant Stability

The influence of some polyols on the stability of enzyme against SDS was also examined by incubating with SDS (0.5% w/v) at 37°C in the presence or absence of polyols (10%, w/v) for 60 minutes, and residual enzyme activity was measured. The data presented in Figure 5(b) showed that 60% residual activity was obtained after incubation with 0.5% w/v SDS with out polyols. But 87.86, 79, 75.54, and 72% residual activity was found with 10% of PEG 4000, PEG 600, mannitol, and glycerol, respectively, after 60 min incubation. From the result, it is clearly indicated that the enzyme stability was significantly found stimulated by polyols. Such type of improvement was also observed in an alkaline protease by the addition of polyols [31, 32]. Therefore, enzyme could be used as a potential candidate for use as a detergent additive without PEG-4000.

#### 3.2.5. Effect of Polyols on Detergent Stability

The detergents stability study was done in the presence of locally available detergents (Tide, Surf Excel, Ariel, and Rin), and results showed that the maximum protease stability was observed with Rin (91%) after incubation with this detergent at 40°C for 1 hr, followed by Tide (89.65%), Ariel (81%), and Surf Excel (31.89%) (data not shown). So, further study was done to evaluate the effect of polyols on detergent stability. In present study, the purified alkaline protease (0.05 mg/mL) was incubated at 40°C using Surf Excel (7 mg/mL) in the presence of various polyols (10%). At every 15 min interval, the residual protease activity was determined up to 1 hour.

The data presented in Figure 5(c) showed that most used polyols improve detergent stability. In the absence of any polyols, enzyme retained 57, 48, 47, and 32% of its initial activity after 15, 30, 45, and 60 min incubation. However, maximum stabilization was found with 10% w/v PEG 4000. Result showed that the enzyme was stable 71, 67, 67, and 64% at 10% of PEG 4000, PEG 600, mannitol, glycerol, respectively after 60 min incubation. Therefore, enzyme along with PEG 4000 may be used as detergents additive.

#### 3.2.6. Evaluation of Washing Performance Using Blood Stain

**Figure 6:**
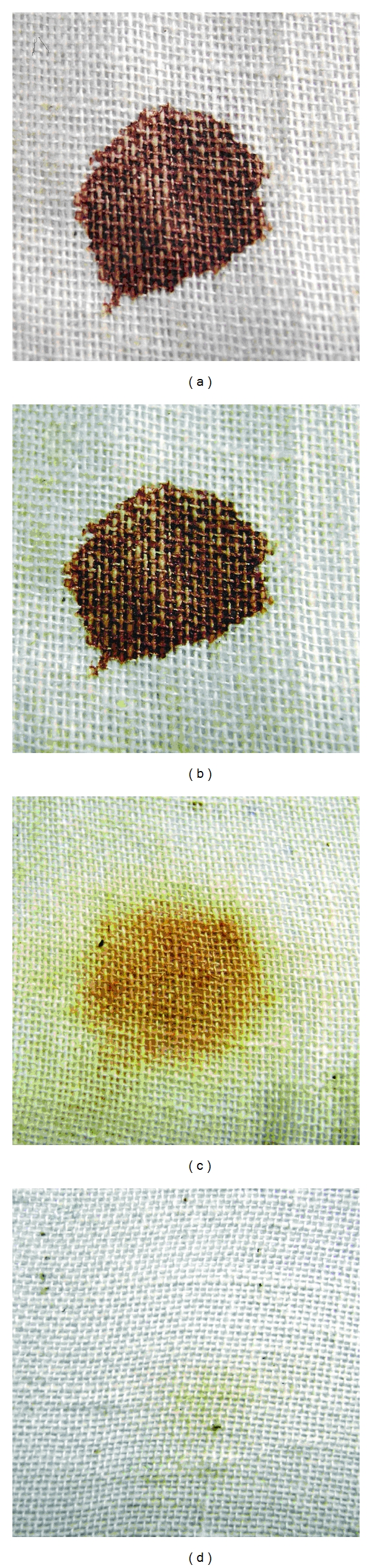
Washing performance analysis of enzyme in the presence of the commercial detergent RIN. (a) Cloth stained with blood, (b) cloth stained with blood and washed with tap water; (b) blood-stained cloth and washed with RIN; (c) blood-stained cloth and washed with RIN added with crude enzyme.

Result showed that the better wash performance was found in combinations of commercial detergents (7 mg/ml) with purified enzyme preparations (2000 U). Result showed that significant removal of the blood stains was found in the presence of the detergent and the protease enzyme (Figure 6) and detergent along with protease enzyme can act synergistically in efficient removal of blood stain. Therefore alkaline protease could be used as detergent additives.

### 3.3. HPLC Analysis of Partially Purified Enzyme

From the HPLC chromatogram of dialysed 60% ammonium sulphate fraction, it was evident that the retention time of our target protein is 1.84 min. The chromatogram represented that this protein is 73.5% pure.

## 4. Conclusion

Due to efficiency and economic concern, the present study was focused on optimization of a variety of culture conditions, for maximal alkaline protease production through microbial fermentation. This is important for obtaining higher alkaline protease production as well as reduction of operating cost in processing. The mathematical analysis has been carried out by standard software, and the procedure followed is user friendly. Taking the characteristics of protease together our protease might be an interesting candidate for the detergent industry in combination with polyols. Use of proteases in detergent can also reduce the volume of detergents which indirectly reduce pollution load in the world.

## Figures and Tables

**Table 1:** Plackett-Burman experimental design for screening of important physical parameters of alkaline protease production by *Bacillus licheniformis NCIM 2042. *

Factor	Name	Low level (−1)	High level (+1)
*A*	Inoculum percentage (%)	2	3
*B*	Temperature (°C)	30	40
*C*	pH	7	8
*D*	Agitation (RPM)	120	180
*E*	Incubation time (h)	72	96

**Table 2:** Experimental range of the five numerical variables studied using rotatable CCD in terms of actual and coded factors.

Factor	Name		Range of variables			
		−*α*(−2.38)	Low (−1)	Mid (0)	High (+1)	+*α* (+2.38)
*A*	Inoculum Percentage (%)	1.3	2	2.5	3	3.7
*B*	Temperature (°C)	23	30	35	40	47
*C*	pH	6.3	7	7.5	8	8.7
*D*	Agitation (RPM)	79	120	150	180	221
*E*	Incubation time (h)	55.5	72	84	96	112.5

**Table 3:** Estimated effect and analysis of variables for protease activity from Plackett-Burman design experiment.

Factor	Name	Studentized effect	Sum square	Percentage of contribution	Standard error	*t*-value	*P* value
*A*	Inoculum percentage (%)	15.92	760.29	2.72	0.34	47.44	<0.0001
*B*	Temperature (°C)	29.35	2585.08	9.26	0.34	87.48	<0.0001
*C*	pH	27.49	2267.32	8.12	0.34	81.93	<0.0001
*D*	Agitation (RPM)	82.11	20228.37	72.46	0.34	244.71	<0.0001
*E*	Incubation time (h)	26.25	2067.06	7.40	0.34	78.23	<0.0001

**Table 4:** Plackett-Burman design for five variables with actual values along with the observed protease activity.

Sl. number	*A*	*B*	*C*	*D*	*E*	Protease activity (U)
1	3	40	7	180	96	257.23
2	2	40	8	120	96	166.03
3	3	30	8	180	72	176.52
4	2	40	7	180	96	275.47
5	2	30	8	120	96	134.25
6	2	30	7	180	72	218.98
7	3	30	7	120	96	147.30
8	3	40	7	120	72	150.14
9	3	40	8	120	72	122.58
10	2	40	8	180	72	219.67
11	3	30	8	180	96	201.53
12	2	30	7	120	72	136.41

**Table 5:** Rotatable CCD matrix for five variables with actual protease activity.

Sl. number	*A*	*B*	*C*	*D*	*E*	Protease activity (U)
1	2	30	7	120	72	132.49
2	3	30	7	120	72	122.78
3	2	40	7	120	72	157.69
4	3	40	7	120	72	152.79
5	2	30	8	120	72	115.42
6	3	30	8	120	72	102.48
7	2	40	8	120	72	136.70
8	3	40	8	120	72	131.90
9	2	30	7	180	72	233.79
10	3	30	7	180	72	215.84
11	2	40	7	180	72	261.54
12	3	40	7	180	72	249.97
13	2	30	8	180	72	213.10
14	3	30	8	180	72	193.58
15	2	40	8	180	72	239.87
16	3	40	8	180	72	223.89
17	2	30	7	120	96	170.54
18	3	30	7	120	96	152.69
19	2	40	7	120	96	200.84
20	3	40	7	120	96	190.84
21	2	30	8	120	96	134.84
22	3	30	8	120	96	116.60
23	2	40	8	120	96	164.75
24	3	40	8	120	96	151.32
25	2	30	7	180	96	242.52
26	3	30	7	180	96	215.75
27	2	40	7	180	96	276.16
28	3	40	7	180	96	257.23
29	2	30	8	180	96	203.78
30	3	30	8	180	96	177.30
31	2	40	8	180	96	236.54
32	3	40	8	180	96	216.43
33	1.3	35	7.5	150	84	286.35
34	3.7	35	7.5	150	84	250.07
35	2.5	23	7.5	150	84	101.30
36	2.5	47	7.5	150	84	174.75
37	2.5	35	6.3	150	84	160.24
38	2.5	35	8.7	150	84	88.85
39	2.5	35	7.5	79	84	103.75
40	2.5	35	7.5	221	84	300.87
41	2.5	35	7.5	150	55.5	131.02
42	2.5	35	7.5	150	112.5	163.67
43	2.5	35	7.5	150	84	273.31
44	2.5	35	7.5	150	84	274.10
45	2.5	35	7.5	150	84	272.82
46	2.5	35	7.5	150	84	132.49
47	2.5	35	7.5	150	84	122.78
48	2.5	35	7.5	150	84	157.69
49	2.5	35	7.5	150	84	152.79
50	2.5	35	7.5	150	84	115.42

**Table 6:** Regression analysis for the production of alkaline protease by *Bacillus licheniformis NCIM 2042 *for quadratic response surface model fitting (ANOVA).

Source	Sum of squares	Degree of freedom	Mean square	Coefficient *t* estimate	Standard error	*F*-value	*P* value prob > *F*	
Model*	181834.9	20	9091.75	—	—	5602.23	<0.0001	Significant
Intercept	—	—	—	271.59	0.45	—	<0.0001	
*A*	2598.525	1	2598.53	−7.75	0.19	1601.18	<0.0001	
*B*	10664.44	1	10664.44	15.69	0.19	6571.31	<0.0001	
*C*	9597.122	1	9597.12	−14.89	0.19	5913.64	<0.0001	
*D*	74093.66	1	74093.66	41.36	0.19	45655.68	<0.0001	
*E*	2104.942	1	2104.94	6.97	0.19	1297.04	<0.0001	
*AB*	77.25183	1	77.25	1.55	0.23	47.60	<0.0001	
*AC*	5.974906	1	5.97	−0.43	0.23	3.68	0.0649	
*AD*	133.704	1	133.70	−2.04	0.23	82.39	<0.0001	
*AE*	92.57182	1	92.57	−1.70	0.23	57.04	<0.0001	
*BC*	8.381578	1	8.38	−0.51	0.23	5.16	0.0306	
*BD*	22.72784	1	22.73	0.84	0.23	14.00	0.0008	
*BE*	95.25979	1	95.26	1.73	0.23	58.70	<0.0001	
*CD*	14.67836	1	14.68	−0.68	0.23	9.04	0.0054	
*CE*	569.8502	1	569.85	−4.22	0.23	351.14	<0.0001	
*DE*	1741.185	1	1741.19	−7.38	0.23	1072.90	<0.0001	
*A* ^2^	19.17653	1	19.18	−0.59	0.17	11.82	0.0018	
*B* ^2^	30951.91	1	30951.91	−23.60	0.17	19072.22	<0.0001	
*C* ^2^	37519.93	1	37519.93	−25.98	0.17	23119.36	<0.0001	
*D* ^2^	8321.356	1	8321.36	−12.24	0.17	5127.53	<0.0001	
*E* ^2^	26782.87	1	26782.87	−21.95	0.17	16503.30	<0.0001	
Residual	47.0635	29	1.62					
Lack of Fit	8.692591	22	0.40			0.07	1	Not significant
Pure Error	38.37091	7	5.48					
Cor Total	181882	49						

*SD: 1.27; Mean: 198.51; R-Squared: 0.9997; Adj R-Squared: 0.9996; C.V.%: 0.64; PRESS: 81.03.

**Table 7:** Validation of quadratic model within the design space.

Number	Inoculum percentage %	Temperature °C	pH	Agitation RPM	Incubation time h	Actual response (U)	Predict response (U)
1	2	35	7	120	72	165.24	168.68
2	2.5	37	7.5	150	72	242.03	244.48
3	3	40	8	160	96	199.17	205.25
4	2.5	30	7.8	180	84	139.65	242.2
5	3	37	7.4	150	74	248.21	247.53
6	2.0	36.6	7.35	180	85	315.28	314.65
